# Aqua­tricarbon­yl(3,5,7-tribromo­tropolonato)rhenium(I) methanol solvate

**DOI:** 10.1107/S1600536808038737

**Published:** 2008-11-22

**Authors:** Marietjie Schutte, Hendrik G. Visser, Andreas Roodt

**Affiliations:** a Department of Chemistry, University of the Free State, PO Box 339, Bloemfontein 9300, South Africa

## Abstract

The title complex, [Re(C_7_H_2_Br_3_O_2_)(CO)_3_(H_2_O)]·CH_3_OH, crystallized as a neutral Re^I^ compound and one methanol solvent mol­ecule in the asymmetric unit. The metal centre is coordinated facially by three carbonyl groups. The bidentate tribromo­tropolanate ligand and a water mol­ecule complete the distorted octahedral coordination around the central metal. Inter­molecular Br⋯O [3.226 (5) Å] and Br⋯Br [3.590 (2) Å] contacts are observed between adjacent mol­ecules. These contacts, together with an array of O—H⋯O, O—H⋯Br and C—H⋯O hydrogen bonds, complete a three-dimensional polymeric network formed between the methanol solvent and the complex.

## Related literature

For a smiliar tribromo­tropolonato Re^I^ structure, see: Schutte *et al.* (2007 ▶). For other related structures, see: Kemp (2006 ▶); Roodt *et al.* (2003 ▶); Wang *et al.* (2003 ▶); Alvarez *et al.* (2007 ▶); Brasey *et al.* (2004 ▶); Gibson *et al.* (1999 ▶); Bochkova *et al.* (1987 ▶); Cheng *et al.* (1988 ▶); Mundwiler *et al.* (2004 ▶). For the synthesis of the precursor, see: Alberto *et al.* (1996 ▶). For synthesis of the tribromo­tropolone ligand, see: Steyl & Roodt (2006 ▶).
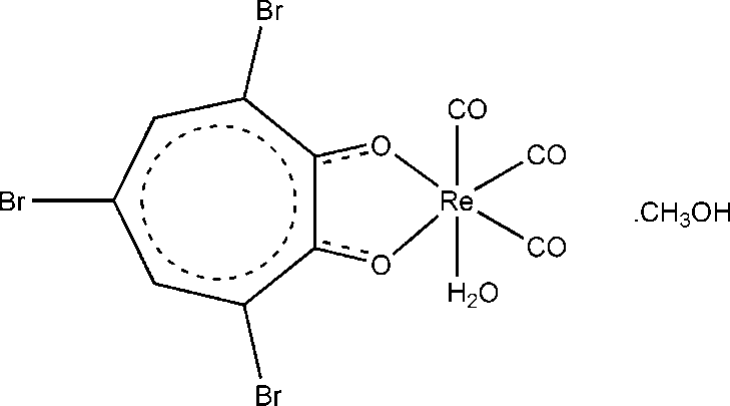


## Experimental

### 

#### Crystal data

[Re(C_7_H_2_Br_3_O_2_)(CO)_3_(H_2_O)]·CH_4_O
*M* *_r_* = 678.1
Triclinic, 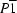

*a* = 9.090 (5) Å
*b* = 9.379 (5) Å
*c* = 10.010 (5) Å
α = 109.569 (5)°
β = 94.285 (5)°
γ = 102.133 (5)°
*V* = 776.3 (7) Å^3^
*Z* = 2
Mo *K*α radiation
μ = 15.58 mm^−1^
*T* = 100 (2) K
0.19 × 0.06 × 0.03 mm

#### Data collection

Bruker APEX diffractometer
Absorption correction: multi-scan (*SADABS*; Bruker, 2004 ▶) *T*_min_ = 0.150, *T*_max_ = 0.626
8673 measured reflections
3599 independent reflections
3018 reflections with *I* > 2σ(*I*)
*R*_int_ = 0.035

#### Refinement

*R*[*F* ^2^ > 2σ(*F* ^2^)] = 0.033
*wR*(*F* ^2^) = 0.079
*S* = 1.05
3599 reflections
207 parameters
H atoms treated by a mixture of independent and constrained refinement
Δρ_max_ = 2.40 e Å^−3^
Δρ_min_ = −2.11 e Å^−3^

### 

Data collection: *APEX2* (Bruker, 2005 ▶); cell refinement: *SAINT-Plus* (Bruker, 2004 ▶); data reduction: *SAINT-Plus*; program(s) used to solve structure: *SHELXS97* (Sheldrick, 2008 ▶); program(s) used to refine structure: *SHELXS97* (Sheldrick, 2008 ▶); molecular graphics: *DIAMOND* (Brandenberg & Putz, 2005 ▶) and *ORTEP-3* (Farrugia, 1999 ▶); software used to prepare material for publication: *SHELXL97*.

## Supplementary Material

Crystal structure: contains datablocks global, I. DOI: 10.1107/S1600536808038737/kj2103sup1.cif

Structure factors: contains datablocks I. DOI: 10.1107/S1600536808038737/kj2103Isup2.hkl

Additional supplementary materials:  crystallographic information; 3D view; checkCIF report

## Figures and Tables

**Table:** 

Re01—C1	1.882 (7)
Re01—C3	1.897 (6)
Re01—C2	1.899 (7)
Re01—O4	2.123 (5)
Re01—O5	2.146 (4)
Re01—O6	2.170 (5)

**Table:** 

O4—Re01—O5	74.07 (16)
O4—Re01—O6	78.93 (19)
O5—Re01—O6	79.17 (18)

**Table 2:** Hydrogen-bond geometry (Å, °)

*D*—H⋯*A*	*D*—H	H⋯*A*	*D*⋯*A*	*D*—H⋯*A*
O6—H6*B*⋯Br1^i^	1.06 (8)	2.68 (8)	3.421 (6)	127 (5)
O6—H6*B*⋯O5^i^	1.06 (8)	1.86 (8)	2.825 (7)	149 (6)
C15—H15⋯O2^ii^	0.93	2.5	3.409 (8)	166
O7—H7⋯O1^iii^	0.82	2.39	2.986 (7)	130
O6—H6*A*⋯O7	0.99 (8)	1.69 (8)	2.665 (7)	167 (7)

Symmetry codes: (i) 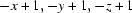; (ii) 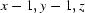; (iii) 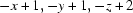.

**Fig. 1.**
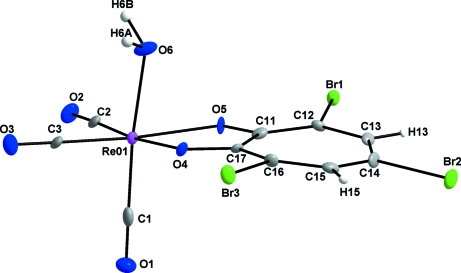
Representation of the title compound, showing the numbering scheme and displacement ellipsoids (50% probability).

## supplementary crystallographic information

### Comment

This structure forms part of an ongoing investigation of the structural and kinetic behaviour of *fac*-Re(CO)_3_ compounds (Schutte *et al.*, 2007; Roodt *et al.*, 2003). The title complex crystallized as a neutral Re^I^ compound and one methanol solvate molecule in the assymetric unit. The Re—CO bond distances are well within the normal range. The Re—O bond distances compare well with the analogous bromido complex (Schutte *et al.*, 2007) and other related structures (Alvarez *et al.*, 2007; Brasey *et al.*, 2004; Gibson *et al.*, 1999; Bochkova *et al.*, 1987; Cheng *et al.*, 1988; Wang *et al.*, 2003). The Re—OH_2_ distance is also comparable to that of related structures (Mundwiler *et al.*, 2004; Kemp, 2006). The small bite angle O4—Re01—O5 might be the reason for the slightly distorted octahedral geometry around the Re^1^ metal centre.

Interesting intermolecular Br···O and Br···Br contacts are observed between adjacent molecules with distances of 3.226 (5) Å between Br1 and O3 and 3.590 (2) Å between Br2 and Br2 of the next molecule. These contacts together with an array of O—H···O, O—H···Br and C—H···O hydrogen bonds (see Table 2), complete a complex three-dimensional polymeric network.

### Experimental

[NEt_4_]_2_[Re(CO)_3_Br_3_] was prepared as described by Alberto *et al.* (1996). 300 mg (0.3894 mmole) of [NEt_4_]_2_[Re(CO)_3_Br_3_] was dissolved in 10 ml of H_2_O at pH 2.2 and stirred for 30 minutes (until dissolved). AgNO_3_ (198 mg, 1.167 mmol) was added to the solution and stirred for 24 h at room temperature. AgBr was formed as a grey precipitate and was filtered off and weighed (0.220 g). Tribromotroplone [151 mg, 0.4514 mmol for synthesis see Steyl & Roodt (2006)] in 2 ml of methanol was added the solution and stirred for 40 h at room temperature. The filtrate was left to stand for a few days and orange plate-like crystals suitable for X-ray diffraction were collected.

### Refinement

The aromatic H atoms were placed in geometrically idealized positions and constrained to ride on its parent atoms with *U*_iso_(H) = 1.2*U*_eq_(C). The highest electron density lies within 1.14 Å from Re. The hydrogen atoms of the coordinated water molecule were determined from a difference Fourier map and their positional parameters freely refined with *U*_iso_(H) = 1.5*U*_eq_(O).

### Crystal data

**Table:**

[Re(C_7_H_2_Br_3_O_2_)(CO)_3_(H_2_O)]·CH_4_O	*Z* = 2
*M**_r_* = 678.1	*F*_000_ = 620
Triclinic, *P*1	*D*_x_ = 2.901 Mg m^−^^3^
Hall symbol: -P 1	Mo *K*α radiation λ = 0.71073 Å
*a* = 9.090 (5) Å	Cell parameters from 3145 reflections
*b* = 9.379 (5) Å	θ = 2.2–28.2º
*c* = 10.010 (5) Å	µ = 15.58 mm^−^^1^
α = 109.569 (5)º	*T* = 100 (2) K
β = 94.285 (5)º	Plate, orange
γ = 102.133 (5)º	0.19 × 0.06 × 0.03 mm
*V* = 776.3 (7) Å^3^	

### Data collection

**Table:**

Bruker APEX diffractometer	*R*_int_ = 0.035
φ and ω scans	θ_max_ = 28.3º
Absorption correction: multi-scan(SADABS; Bruker, 2004)	θ_min_ = 2.2º
*T*_min_ = 0.150, *T*_max_ = 0.626	*h* = −8→12
8673 measured reflections	*k* = −11→12
3599 independent reflections	*l* = −13→10
3018 reflections with *I* > 2σ(*I*)	

### Refinement

**Table:**

Refinement on *F*^2^	H atoms treated by a mixture of independent and constrained refinement
Least-squares matrix: full	*w* = 1/[σ^2^(*F*_o_^2^) + (0.0385*P*)^2^] where *P* = (*F*_o_^2^ + 2*F*_c_^2^)/3
*R*[*F*^2^ > 2σ(*F*^2^)] = 0.033	(Δ/σ)_max_ < 0.001
*wR*(*F*^2^) = 0.079	Δρ_max_ = 2.41 e Å^−^^3^
*S* = 1.05	Δρ_min_ = −2.11 e Å^−^^3^
3599 reflections	Extinction correction: none
207 parameters	

### Special details

**Table:**

Geometry. All e.s.d.'s (except the e.s.d. in the dihedral angle between two l.s. planes) are estimated using the full covariance matrix. The cell e.s.d.'s are taken into account individually in the estimation of e.s.d.'s in distances, angles and torsion angles; correlations between e.s.d.'s in cell parameters are only used when they are defined by crystal symmetry. An approximate (isotropic) treatment of cell e.s.d.'s is used for estimating e.s.d.'s involving l.s. planes.

### Fractional atomic coordinates and isotropic or equivalent isotropic displacement parameters (Å^2^)

**Table:**

	*x*	*y*	*z*	*U*_iso_*/*U*_eq_	
Re01	0.54228 (3)	0.51098 (3)	0.75340 (3)	0.00853 (8)	
Br1	0.46576 (7)	0.05513 (7)	0.28520 (6)	0.01185 (14)	
O4	0.3428 (5)	0.3587 (5)	0.7724 (4)	0.0107 (9)	
C11	0.3852 (7)	0.1903 (7)	0.5557 (7)	0.0098 (13)	
C2	0.7128 (8)	0.6389 (7)	0.7149 (7)	0.0141 (8)	
C15	0.1120 (7)	−0.0432 (8)	0.6174 (7)	0.0130 (13)	
H15	0.0347	−0.093	0.6543	0.016 (19)*	
C3	0.5502 (8)	0.6908 (8)	0.9164 (7)	0.0141 (8)	
C17	0.2988 (7)	0.2213 (7)	0.6772 (6)	0.0079 (12)	
O5	0.4976 (5)	0.3042 (5)	0.5642 (4)	0.0096 (9)	
C16	0.1735 (7)	0.1137 (7)	0.6939 (6)	0.0100 (13)	
C12	0.3491 (7)	0.0514 (7)	0.4343 (6)	0.0084 (12)	
C13	0.2524 (7)	−0.0921 (7)	0.4077 (7)	0.0101 (13)	
H13	0.2554	−0.1698	0.3217	0.012 (18)*	
C14	0.1514 (7)	−0.1368 (7)	0.4909 (7)	0.0135 (13)	
Br2	0.04885 (8)	−0.35228 (8)	0.42285 (7)	0.01741 (15)	
Br3	0.08290 (7)	0.19489 (8)	0.85829 (7)	0.01438 (15)	
O2	0.8201 (5)	0.7193 (5)	0.6960 (5)	0.0181 (11)	
O3	0.5530 (6)	0.8006 (6)	1.0157 (5)	0.0191 (11)	
O1	0.7678 (6)	0.4158 (6)	0.9272 (5)	0.0221 (11)	
C1	0.6804 (8)	0.4526 (8)	0.8620 (7)	0.0153 (14)	
O7	0.1793 (6)	0.6493 (6)	0.8032 (5)	0.0186 (11)	
H7	0.1438	0.5913	0.8449	0.028*	
C4	0.1962 (8)	0.8155 (7)	0.8974 (7)	0.0141 (8)	
H4C	0.2448	0.833	0.992	0.021*	
H4A	0.0974	0.8365	0.9024	0.021*	
H4B	0.2572	0.8837	0.8583	0.021*	
O6	0.3692 (6)	0.5548 (6)	0.6216 (5)	0.0214 (11)	
H6A	0.295 (9)	0.599 (9)	0.681 (8)	0.032*	
H6B	0.406 (9)	0.640 (9)	0.575 (8)	0.032*	

### Atomic displacement parameters (Å^2^)

**Table:**

	*U*^11^	*U*^22^	*U*^33^	*U*^12^	*U*^13^	*U*^23^
Re01	0.00886 (14)	0.00633 (14)	0.00918 (13)	−0.00064 (10)	0.00140 (9)	0.00280 (10)
Br1	0.0139 (3)	0.0099 (3)	0.0110 (3)	0.0015 (3)	0.0039 (2)	0.0033 (2)
O4	0.016 (3)	0.006 (2)	0.010 (2)	0.001 (2)	0.0039 (18)	0.0042 (18)
C11	0.007 (3)	0.012 (3)	0.015 (3)	0.005 (3)	0.004 (2)	0.008 (3)
C2	0.024 (2)	0.0071 (19)	0.0122 (17)	0.0075 (18)	0.0005 (15)	0.0033 (15)
C15	0.010 (3)	0.017 (4)	0.014 (3)	0.002 (3)	−0.002 (2)	0.009 (3)
C3	0.024 (2)	0.0071 (19)	0.0122 (17)	0.0075 (18)	0.0005 (15)	0.0033 (15)
C17	0.010 (3)	0.007 (3)	0.010 (3)	0.001 (3)	0.000 (2)	0.006 (2)
O5	0.010 (2)	0.004 (2)	0.011 (2)	−0.0030 (19)	0.0036 (17)	−0.0002 (17)
C16	0.011 (3)	0.012 (3)	0.008 (3)	0.002 (3)	0.003 (2)	0.005 (3)
C12	0.006 (3)	0.013 (3)	0.007 (3)	0.001 (3)	0.001 (2)	0.005 (2)
C13	0.004 (3)	0.010 (3)	0.014 (3)	0.000 (3)	−0.002 (2)	0.004 (3)
C14	0.010 (3)	0.007 (3)	0.019 (3)	−0.004 (3)	−0.004 (3)	0.004 (3)
Br2	0.0181 (4)	0.0091 (3)	0.0225 (3)	−0.0015 (3)	0.0040 (3)	0.0052 (3)
Br3	0.0123 (3)	0.0144 (3)	0.0140 (3)	−0.0005 (3)	0.0057 (3)	0.0036 (3)
O2	0.016 (3)	0.015 (3)	0.025 (3)	0.001 (2)	0.010 (2)	0.011 (2)
O3	0.017 (3)	0.017 (3)	0.018 (2)	0.005 (2)	0.003 (2)	−0.001 (2)
O1	0.022 (3)	0.025 (3)	0.020 (3)	0.008 (2)	−0.001 (2)	0.010 (2)
C1	0.020 (4)	0.009 (3)	0.012 (3)	0.000 (3)	0.003 (3)	−0.001 (3)
O7	0.025 (3)	0.021 (3)	0.022 (3)	0.013 (2)	0.008 (2)	0.018 (2)
C4	0.024 (2)	0.0071 (19)	0.0122 (17)	0.0075 (18)	0.0005 (15)	0.0033 (15)
O6	0.025 (3)	0.025 (3)	0.025 (3)	0.013 (3)	0.008 (2)	0.018 (2)

### Geometric parameters (Å, °)

**Table:**

Re01—C1	1.882 (7)	C3—O3	1.162 (8)
Re01—C3	1.897 (6)	C17—C16	1.415 (8)
Re01—C2	1.899 (7)	C16—Br3	1.895 (6)
Re01—O4	2.123 (5)	C12—C13	1.372 (9)
Re01—O5	2.146 (4)	C13—C14	1.378 (9)
Re01—O6	2.170 (5)	C13—H13	0.93
Br1—C12	1.899 (6)	C14—Br2	1.900 (6)
O4—C17	1.278 (7)	O1—C1	1.168 (8)
C11—O5	1.289 (7)	O7—C4	1.495 (8)
C11—C12	1.408 (9)	O7—H7	0.82
C11—C17	1.477 (8)	C4—H4C	0.96
C2—O2	1.171 (8)	C4—H4A	0.96
C15—C16	1.379 (9)	C4—H4B	0.96
C15—C14	1.398 (9)	O6—H6A	0.99 (8)
C15—H15	0.93	O6—H6B	1.06 (8)
			
C1—Re01—C3	89.5 (3)	C16—C17—C11	125.5 (6)
C1—Re01—C2	87.8 (3)	C11—O5—Re01	117.1 (4)
C3—Re01—C2	85.0 (3)	C15—C16—C17	131.3 (6)
C1—Re01—O4	96.2 (2)	C15—C16—Br3	113.9 (5)
C3—Re01—O4	99.6 (2)	C17—C16—Br3	114.6 (4)
C2—Re01—O4	173.9 (2)	C13—C12—C11	131.5 (6)
C1—Re01—O5	96.7 (2)	C13—C12—Br1	113.1 (4)
C3—Re01—O5	171.5 (2)	C11—C12—Br1	115.2 (5)
C2—Re01—O5	100.9 (2)	C12—C13—C14	128.9 (6)
O4—Re01—O5	74.07 (16)	C12—C13—H13	115.6
C1—Re01—O6	174.3 (3)	C14—C13—H13	115.6
C3—Re01—O6	94.2 (2)	C13—C14—C15	128.3 (6)
C2—Re01—O6	96.8 (2)	C13—C14—Br2	115.9 (5)
O4—Re01—O6	78.93 (19)	C15—C14—Br2	115.8 (5)
O5—Re01—O6	79.17 (18)	O1—C1—Re01	178.7 (6)
C17—O4—Re01	118.0 (4)	C4—O7—H7	109.5
O5—C11—C12	120.1 (5)	O7—C4—H4C	109.5
O5—C11—C17	115.0 (5)	O7—C4—H4A	109.5
C12—C11—C17	124.9 (6)	H4C—C4—H4A	109.5
O2—C2—Re01	177.7 (6)	O7—C4—H4B	109.5
C16—C15—C14	128.0 (6)	H4C—C4—H4B	109.5
C16—C15—H15	116	H4A—C4—H4B	109.5
C14—C15—H15	116	Re01—O6—H6A	110 (4)
O3—C3—Re01	179.1 (6)	Re01—O6—H6B	117 (4)
O4—C17—C16	119.0 (5)	H6A—O6—H6B	102 (6)
O4—C17—C11	115.4 (6)		

### Hydrogen-bond geometry (Å, °)

**Table:**

*D*—H···*A*	*D*—H	H···*A*	*D*···*A*	*D*—H···*A*
O6—H6B···Br1^i^	1.06 (8)	2.68 (8)	3.421 (6)	127 (5)
O6—H6B···O5^i^	1.06 (8)	1.86 (8)	2.825 (7)	149 (6)
C15—H15···O2^ii^	0.93	2.5	3.409 (8)	166
O7—H7···O1^iii^	0.82	2.39	2.986 (7)	130
O6—H6A···O7	0.99 (8)	1.69 (8)	2.665 (7)	167 (7)

Symmetry codes: (i) −*x*+1, −*y*+1, −*z*+1; (ii) *x*−1, *y*−1, *z*; (iii) −*x*+1, −*y*+1, −*z*+2.
